# Transcriptional Correlates of Disease Outcome in Anticoagulant-Treated Non-Human Primates Infected with Ebolavirus

**DOI:** 10.1371/journal.pntd.0003061

**Published:** 2014-07-31

**Authors:** Sara Garamszegi, Judy Y. Yen, Anna N. Honko, Joan B. Geisbert, Kathleen H. Rubins, Thomas W. Geisbert, Yu Xia, Lisa E. Hensley, John H. Connor

**Affiliations:** 1 Bioinformatics Program, Boston University, Boston, Massachusetts, University of America; 2 Department of Microbiology, School of Medicine, Boston University, Boston, Massachusetts, University of America; 3 National Emerging Infectious Diseases Laboratories, Boston University, Boston, Massachusetts, University of America; 4 U.S. Army Medical Research Institute of Infectious Diseases, Fort Detrick, Frederick, Maryland, United States of America; 5 Department of Microbiology and Immunology, University of Texas Medical Branch, Galveston, Texas, United States of America; 6 National Aeronautics and Space Administration, Houston, Texas, United States of America; 7 Department of Bioengineering, McGill University, Montreal, Canada; 8 Integrated Research Facility at Fort Detrick, National Institute of Allergy and Infectious Diseases, National Institutes of Health, Fort Detrick, Frederick, Maryland, United States of America; Centers for Disease Control and Prevention, United States of America

## Abstract

Ebola virus (EBOV) infection in humans and non-human primates (NHPs) is highly lethal, and there is limited understanding of the mechanisms associated with pathogenesis and survival. Here, we describe a transcriptomic analysis of NHPs that survived lethal EBOV infection, compared to NHPs that did not survive. It has been previously demonstrated that anticoagulant therapeutics increase the survival rate in EBOV-infected NHPs, and that the characteristic transcriptional profile of immune response changes in anticoagulant-treated NHPs. In order to identify transcriptional signatures that correlate with survival following EBOV infection, we compared the mRNA expression profile in peripheral blood mononuclear cells from EBOV-infected NHPs that received anticoagulant treatment, to those that did not receive treatment. We identified a small set of 20 genes that are highly confident predictors and can accurately distinguish between surviving and non-surviving animals. In addition, we identified a larger predictive signature of 238 genes that correlated with disease outcome and treatment; this latter signature was associated with a variety of host responses, such as the inflammatory response, T cell death, and inhibition of viral replication. Notably, among survival-associated genes were subsets of genes that are transcriptionally regulated by (1) CCAAT/enhancer-binding protein alpha, (2) tumor protein 53, and (3) megakaryoblastic leukemia 1 and myocardin-like protein 2. These pathways merit further investigation as potential transcriptional signatures of host immune response to EBOV infection.

## Author Summary

Infection of humans and non-human primates (NHPs) with Ebola virus (EBOV) can cause viral hemorrhagic fever, an acute systemic illness which can lead to death. The high case fatality rates (25%–90%) make EBOV a virus of significant concern from a biodefense perspective. To date, there are no FDA-approved post-exposure treatments for human use, and there are no standard assays to predict how infected individuals will fare after becoming infected. We have analyzed how circulating immune cells respond to EBOV infection under conditions where NHPs either survive viral infection, or succumb to it. This analysis identified genes that are correlated with, and predictive of, survival following lethal EBOV infection in NHPs. Our results demonstrate that small gene sets and transcriptional regulatory networks can be used to identify individual markers associated with survival following EBOV infection.

## Introduction

Ebola virus (EBOV; *Filoviridae* [1]) infection of humans and non-human primates (NHPs) can cause viral hemorrhagic fever, an acute systemic illness characterized by fever, bleeding diathesis, fulminant shock, and death [2]. Although several studies have identified candidate therapeutics that may mitigate the effects of EBOV infection [3]–[12], there are currently no FDA-approved post-exposure treatments for human use. Additionally, despite extensive research on EBOV pathogenesis [13]–[15], lifecycle [16], and interactions with the host [17]–[19], there are no standard biomarkers to predict host immune response to EBOV infection, nor are there biomarkers of drug efficacy or survival following treatment. Here, we investigated the hypothesis that there are signatures of gene expression associated with survival in EBOV-infected, anticoagulant-treated NHPs.

Immune response pathways play a key role in EBOV pathogenesis. Infection is characterized by up-regulation of inflammatory mediators such as cytokines and chemokines, interleukins, interferon-inducible proteins, and tumor necrosis factor alpha (TNFα) [20]–[25]. In addition, EBOV infection is associated with an early loss of lymphocytes [26], [27] and the dysregulation of coagulopathy. This dysregulation of coagulation and subsequent hemorrhage are characteristic of EBOV infection [14], [15], and may be due to the fact that immune mediators are over-expressed by monocytes and macrophages, which, along with dendritic cells, are primary targets for infection [13], [28], [29]. High-throughput microarray studies of the immune response of NHPs to EBOV infection have identified dramatic and early changes in host transcription of genes related to interferon response, cytokine signaling, and apoptosis [22]. Similarly, studies of endothelial cells suggest that accumulation of cytokines and other pro-inflammatory factors can contribute to the observed pathologies of EBOV infection [14]. Previous studies of clinical samples from humans infected with Sudan virus during the 2000–2001 outbreak, suggest that hemorrhagic symptoms and death may be associated with acute phase proteins and coagulation factors [25].

It has been demonstrated that anticoagulant therapeutics have a positive effect on the outcome of EBOV disease [7], [9]. Notably, approximately 33% of EBOV-infected NHPs treated with anticoagulants, such as recombinant nematode anticoagulant protein c2 (rNAPc2) [7], and recombinant human activated protein C (rhAPC) [9] survived a 100% fatal EBOV infection model (Table 1). Animals responding to anticoagulant treatment had lower plasma viremia levels and attenuation of the pro-inflammatory and pro-coagulant responses in both studies [7], [9], suggesting that these indicators could be markers of increased survival. However, these late-stage markers could not identify whether there were early transcriptional changes that were associated with survival. In addition, these results are limited in scope to individual gene and protein assays, which cannot assess the host immune response to infection from a global, transcriptional viewpoint [7], [9]. To identify early-stage transcriptional changes, we analyzed an existing microarray dataset that examined the host gene expression in anticoagulant-treated NHPs. We used this dataset to identify critical transcriptional changes that differentiate between surviving and non-surviving NHPs following EBOV infection. These results provide critically distinct assessments of the host immune response to EBOV infection by identifying not only global changes in transcription, but also transcription factor activities associated with survival.

**Table 1:** Summary of methods and results from previous publications.

	Dataset 1		Dataset 2	
**Study**	EBOV infected NHPs, treated with rNAPc2 [7]		EBOV infected NHPs, treated with rhAPC (Xigris) [9]	
**Survival**	33% (3/9 NHPs receiving rNAPc2). Mean time to death: 8.3 days (EBOV only); 11.7 days (EBOV+rNAPc2).		18% (2/11 NHPs receiving rhAPC). Mean time to death: 8.3 days (EBOV only); 12.6 days (EBOV+rhAPC).	
**Number of Samples Used in This Study** *				
	Survivor−rNAPc2	–	Survivor−rhAPC	–
	Survivor+rNAPc2	2	Survivor+rhAPC	2
	Non-Survivor−rNAPc2	2	Non-Survivor−rhAPC	2
	Non-Survivor+rNAPc2	6	Non-Survivor+rhAPC	9

This table briefly describes the infectious agent, treatment, methods, and results previously been described in Geisbert *et al.* [7] and Hensley *et al.* [9].

EBOV: Zaire Ebolavirus. NHP: non-human primate. rNAPc2: recombinant nematode anticoagulant protein c2. rhAPC: recombinant human activated protein C.

* Remaining animals did not have samples available for cDNA microarray analysis.

We analyzed the transcriptional profiles of peripheral blood mononuclear cell samples taken from EBOV-infected NHPs treated with either rNAPc2 or rhAPC, as described previously [7], [9], [24]. We investigated the hypothesis that gene expression patterns are associated with survival of EBOV challenge following anticoagulant treatment. A previous study assessed the global transcriptional response of NHPs to EBOV infection, but was unable to identify survival-associated profiles, due to a lack of anticoagulant treatment [22]. Previously, we assessed the global transcriptional response in the context of anticoagulant treatment, but did not seek to identify upstream transcriptional regulators associated with survival [24]. Here, we used a new method of analysis to identify gene sets which are associated with, and predictive of, survival following post-infection treatment. This approach identified two sets of statistically significant genes that distinguish between surviving and non-surviving NHPs. One, a minimal set of 20 genes, showed good discrimination between survivors and non-survivors, but provided little insight into signaling pathways that might be correlated with survival. The second, a larger set of 238 genes, identified a number of genes that were functionally controlled by common transcription factors that have not been previously associated with EBOV infection in NHPs.

## Materials and Methods

### Animal experiments and ethics statement

The datasets for this study were collected from previously published results studying the affect of anticoagulant therapeutics (rhAPC and rNAPc2) on the immune response of non-human primates (NHPs) to lethal Ebola virus (H.sapiens-tc/COD/1995/Kikwit-9510621; EBOV). Virus was passaged twice through VeroE6, and once through Vero cells, prior to use. Additional experimental methods and results of the rhAPC and rNAPc2 studies have previously been described in Geisbert *et al.* [7] and Hensley *et al.* [9], respectively (Table 1). The cumulative dataset used in this study includes 23 rhesus macaques: 4 untreated controls, 8 rNAPc2-treated NHPs, and 11 rhAPC-treated NHPs (Table 2). A total of 4 NHPs survived lethal challenge with EBOV virus (2 rNAPc2-treated and 2 rhAPC-treated).

**Table 2:** Summary of samples used in this study.

Group	EBOV Only				EBOV-infected, Treated Non-Survivor					EBOV-infected, Treated Survivor			
Day/Sample	1	2	3	4	1	2	3	4	5	1	2	3	4
−8			•	•		•		•	•	•			•
0	•	•		•	•	•	•			•	•	•	
3	•	•	•		•		•	•	•	•	•	•	•
6	•	•	•	•	•	•	•	•		•	•	•	•
**rNAPc2**	−	−	−	−	+	−	−	−	−	+	+	−	−
**rhAPC**	−	−	−	−	−	+	+	+	+	−	−	+	+
**Designation**	CH78	CH52	DB7U	GTX	AXX	L201-2	CH73	CL4R	94E136	AA016	CH04	DB1D	DB1L
**Day of Death:**	9	8	8	8	8	9	7	7	8	−	−	−	−

This table depicts the samples (dots) used in this study. Samples are grouped vertically based on treatment, then by individual animal. Samples are also grouped horizontally by day pre- and post-infection. Summary information includes applied treatment (if any), and date of death for each animal. Designations are from the original publications, Geisbert *et al.* [7] and Hensley *et al.* [9].

EBOV: Zaire Ebolavirus. rNAPc2: recombinant nematode anticoagulant protein c2. rhAPC: recombinant human activated protein C.

Animal research for these previously published studies was conducted at the United States Army Medical Research Institute for Infectious Diseases, in compliance with the Animal Welfare Act and other federal statutes and regulations relating to animals and experiments involving animals, and adheres to the principles stated in the *Guide for the Care and Use of Laboratory Animals*, National Research Council, 1996. The facility is fully accredited by the Association for Assessment and Accreditation of Laboratory Animal Care, International. These experiments and procedures were approved by the USAMRIID Institutional Animal Care and Use Committee (IACUC).

### Sample preparation and microarray processing

Experimental methods for RNA processing and DNA microarray preparation have been previously described in Yen *et al.* [24]. Microarrays were analyzed in R, using the LIMMA package in Bioconductor [30]–[32] and processed as follows: (i) background correction was done using the *subtract* method [33]; (ii) within-array normalization was done using the *loess* method [34]; (iii) log ratio and log intensity values were calculated; (iv) array control probes were removed from the dataset; and, (v) the data were zero-transformed within each animal using baseline (pre-infection) sample; in the case of multiple pre-infection samples, Day 0 was used (Figure S1). The raw microarray dataset was deposited in NCBI's Gene Expression Omnibus (GEO; [35]) database (Accession: GSE24943; [24]).

We organized the microarrays into three groups based on NHP response to anticoagulant drug treatment: (i) EBOV-infected NHPs that did not receive anticoagulant treatment (“EBOV Only”; EO); (ii) anticoagulant-treated NHPs that survived EBOV infection (“EBOV infected, Treated Survivors”; ETS); and, (iii) anticoagulant-treated NHPs that did not respond to treatment and did not survive EBOV infection (“EBOV infected, Treated Non-Survivors”; ETNS), which were characterized by a mean time to death indistinguishable from untreated NHPs, *i.e.* animals died prior to Day 10 post-infection. A fourth group, characterized by treated, non-surviving NHPs with a mean time to death greater than the untreated controls, was excluded because any results would have been uninformative with regard to survival or treatment-specific transcriptional signatures. We limited our microarrays to Days 3 and 6 post-infection, because these timepoints were available for all treatment groups. A total of 23 arrays were included in the comparison: 4 arrays for each treatment group on both Day 3 and Day 6, except for the EO group on Day 3, which only had 3 samples (Table 2).

### Identification of a minimal survival-associated gene set

To identify the minimal number of genes which distinguish survivors from non-survivors, we grouped the “EBOV Only” and “EBOV infected, Treated Non-Survivors” groups together into one cumulative “Non-Survivor” (NS) group. We compared survivors against non-survivors on Day 3, Day 6, and Days 3 and 6 together (Figure S1). Gene expression was averaged within each treatment group for individual probes, and the difference in mean expression (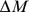) for individual probes was calculated as follows:
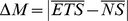
where 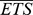 is the mean expression in survivors (ETS), and 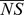 is the mean expression in non-survivors (NS). The probe (and its corresponding gene) is considered biologically relevant if, in at least one of the three comparisons, it meets the following criteria: (i) statistical significance (Student's t-test, unequal variance; *P*≤0.05); (ii) large magnitude (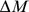
≥2); and (iii) the differences in individual values across a treatment group are in agreement with the difference in means (within-group agreement).

### Identification of general survival-associated transcriptional profile

To determine whether there is a general transcriptional profile associated with survival, we analyzed Day 3 and 6 arrays in EBOV-infected survivors and compared them against NHPs that did not survive infection using six comparisons: the ETS group to the ETNS group, and the ETS group to the EO group, at three time points: Day 3, Day 6, and Days 3 and 6 together (Figure S1). Gene expression was averaged and the difference in mean expression (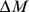) for individual probes is calculated as follows:
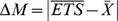
where 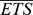 is the mean expression in survivors (ETS), and 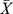 is the mean expression in either of the two non-survivor groups (ETNS or EO). The probe (and its corresponding gene) was considered biologically relevant if, in at least one of the six comparisons, it met the previously described criteria.

### Statistical analysis and validation

The correlation between identified genes and survival was evaluated for statistical accuracy using a permutation test of hierarchical clustering. Expression values for each gene were randomly permutated among arrays (1000 trials); for each trial, complete linkage hierarchical clustering was used to evaluate whether the gene list separated NHPs which survived infection from NHPs that did not survive infection (Pearson correlation coefficient). Clustering was completed using PyCluster [36] and Cluster 3.0 [37]. Heatmaps and dendrograms were generated using Java TreeView [38], and networks were rendered in Cytoscape [39]. The classification ability of each gene set was evaluated using leave-one out cross-validation and receiver operating characteristic (ROC) curves. Classification was evaluated by comparing the sum of normalized distances of the left-out sample to the mean of the survivor and non-survivor groups, for each gene in the gene set. The area under the curve (AUC) was used to evaluate performance of each gene set.

Biologically and statistically relevant genes were probed for functional annotation, pathways, and upstream transcriptional regulators using the Ingenuity Pathway Analysis suite of software (IPA; Ingenuity Systems). We confirmed the expression profile of a subset of our gene set using two comparisons: (1) an independently derived microarray dataset which examined changes in gene expression in EBOV-infected NHPs without anticoagulant treatment; and, (2) reverse transcription-PCR. We examined expression over the course of infection using the following equation:
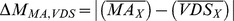
Where 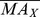 is the mean expression in our dataset on Day 3 or Day 6, and 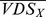 is the mean expression in the “Validation Dataset”, either the second microarray dataset or the RT-PCR, on Day 3 or Days 5/6. We consider “complete agreement” to be a case where the direction of expression in our dataset and the validation dataset are the same (*e.g.* both up-regulated); “minor disagreement” is a case where the direction of expression is opposing in the microarray and validation datasets, but is within 1 log_2_ fold change of difference, and therefore not significantly different; and, “major disagreement” is a case where the direction of expression in the microarray and validation datasets are opposing and significant in magnitude (>1). We evaluated whether the RT-PCR results reflect similar trends in expression, compared to the microarray data, by calculating the percentage of “complete agreement” or “minor disagreement” cases.

We compared the 245 probes from our gene set to the second microarray, for EBOV-infected NHPs that did not receive anticoagulant treatment. Of the 245 probes in our gene set, only 182 were available on the second microarray for confirmation (74.3%). When comparing the two microarray datasets, an average of 132.5 probes (72.8%) were in complete agreement or minor disagreement regarding the changes in expression from baseline to Day 3 or Days 5/6. Of the remaining probes, the majority were cases in which one microarray dataset was not differentially expressed but the other was, or vice versa; there were only 8 cases of significant and opposing disagreement between the two microarray datasets (4.4%).

We also confirmed a subset of our gene set using RT-PCR. Of the 245 probes of interest, we tested 56 genes that were associated with the upstream transcriptional regulators we identified previously; of these 56 genes, 45 passed the quality testing. For EBOV-infected NHPs that did not receive anticoagulant treatment (“EBOV Only”), an average of 34.5 probes (76.7%) were either in complete agreement or minor disagreement with respect to expression on Days 3 and 6. For EBOV-infected, anticoagulant-treated NHPs that did not survive (“EBOV infected, Treated Non-Survivors”), an average of 34 (75.6%) probes were either in complete agreement or minor disagreement. Finally, for EBOV-infected, anticoagulant-treated NHPs that survived infection (“EBOV infected, Treated Survivors”), an average of 37.8 (83.9%) probes were either in complete agreement or minor disagreement between the microarray dataset and RT-PCR.

## Results

### Identification of a minimal survival-associated gene set in EBOV-infected, anticoagulant-treated NHPs

Previous studies have reported that NHPs exhibit strong transcriptional changes in response to EBOV infection [22], [24]. We were interested in determining if these transcriptional changes were altered by anticoagulant treatment; specifically, we were interested to determine if there were any transcriptional correlates associated with survival of EBOV infection.

To determine the minimal number of genes which were associated with survival, we analyzed changes in mRNA expression in EBOV-infected NHPs with and without anticoagulant treatment. Previously published data was used to build a cumulative group of 23 EBOV-infected NHPs ([7], [9]; Table 1): 4 EBOV-infected controls that did not receive anticoagulant treatment, 8 EBOV-infected NHPs that were treated with rNAPC2, and 11 EBOV-infected NHPs that were treated with rhAPC (Table 2). We organized the samples into three groups: (i) EBOV-infected, untreated non-survivors (“EBOV Only”; EO); (ii) EBOV-infected, anticoagulant-treated survivors (“EBOV-infected, Treated Survivors”; ETS); and, (iii) EBOV-infected, anticoagulant-treated, non-survivors (“EBOV-infected, Treated Non-Survivors”; ETNS), which were characterized by a mean time to death indistinguishable from untreated NHPs. In order to identify a set of probes which were associated with survival and not just treatment responses, we compared the gene expression of NHPs that survived EBOV infection (ETS) to those that did not survive (“Non-Survivors”, NS; a combination of the EO and ETNS groups) on Days 3 and 6 post-infection. Probes were considered of interest if the difference in expression between groups was statistically significant (Student's t-test, unequal variance; *P*≤0.05), with a large change in transcriptional magnitude (ΔM≥2), and had within-group coherence (see *Materials and Methods*).

**Figure 1:**
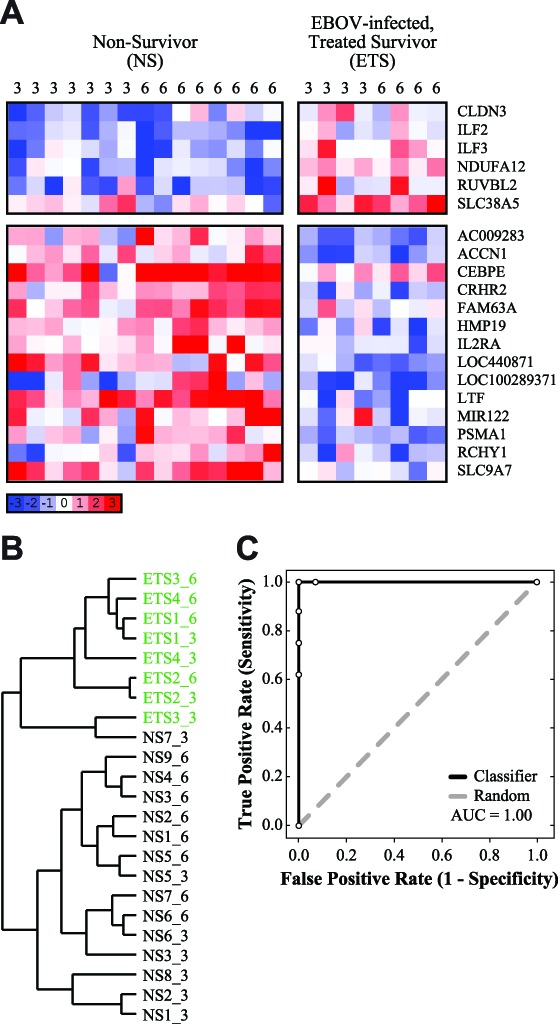
Identification of a small gene set is sufficient for classification of survival in EBOV-infected, anticoagulant-treated NHPs. (**A**) A heatmap depicting the gene expression values of 20 genes which were found to distinguish between “EBOV-infected, Treated Survivors” (ETS) and Non-Survivors (“EBOV Only” and “EBOV-infect, Treated Non-Survivors”; see *Materials and Methods*). The days post-infection are labeled at the top of each column. Blue indicates down-regulation, and red indicates up-regulation, of genes compared to pre-infection baseline, white indicates no change in expression (scale indicated). Gene names are listed to the right. (**B**) A dendrogram depicting hierarchical clustering of individual samples, using the set of 20 genes (permutation test, one-tailed *P*<0.0001). ETS samples are indicated in green; samples are labeled “groupX_Y”, where X is the sample number and Y is the days post-infection. (**C**) A ROC curve depicting how accurately this gene set can classify survivors and non-survivors, based on leave-one-out cross-validation (AUC = 1.00). This gene set is indicated in black, and a random ROC curve is indicated in grey.

We identified a total of 20 unique probes which differentiated between the NHPs that survived EBOV infection (ETS) and those that did not (NS). These probes corresponded to 16 annotated genes, 3 genetic loci, and 1 microRNA (Figure 1). There are two obvious patterns of differentially expressed genes which exhibit significant and opposing regulation in the two groups (Figure 1A): (i) 6 genes have higher expression values in survivors compared to non-survivors (CLDN3, ILF2, ILF3, NDUFA12, RUVBL2, and SLC38A5); and, (ii) 10 genes have lower expression values in survivors compared to non-survivors (ACCN1, CEBPE, CRHR2, FAM63A, HMP19, IL2RA, LTF, PSMA1, RCHY1, and SLC9A7). Finally, the genetic loci (AC009283, LOC100289371, and LOC440871) and microRNA (miR-122) also appear to be down-regulated in survivors compared to non-survivors.

Due to the minimal size of the gene set, it is difficult to perform functional enrichment or pathway analysis; however, identification of individual gene traits and annotation is useful for elucidating how individual genes may be associated with viral infection. For example, over half of these genes have been previously associated with viral infection or replication, or have been found to physically interact with viral proteins (*e.g.* CLDN3 [40]; CRHR2 [41]; ILF2 [42], [43]; ILF3 [44], [45]; LTF [46]–[50]; miR-122 [51]; RCHY1 [52]; and, RUVBL2 [53]). Importantly, none of these genes have been linked to EBOV infection, with the exception of ILF3, of which an isoform called DRBP76 is known to bind EBOV protein VP35 [54]. Although miR-122 has not been associated with EBOV infection, it is a known positive regulator of the replication of hepatitis C virus, another RNA virus [55], [56]. Additionally, several of the genes associated with survival encode for transport and membrane proteins (*e.g.* ACCN1, CLDN3, HMP19, NDUFA12, SLC9A7, and SLC38A5), suggesting that small-molecule transport proteins may play a role in survival of EBOV challenge. Several gene products are also associated with immune response or inflammatory response (*e.g.* CRHR2, IL2RA, LTF, and PSMA1), which is consistent with previously published studies investigating the effects of EBOV on gene expression [21], [22], [24].

Having identified a minimal survival-associated gene set, we were interested in determining whether it could accurately distinguish between survivors and non-survivors. Hierarchical clustering is a common way to use the characteristics of a gene set to determine if datasets are similar or dissimilar. We used hierarchical clustering to demonstrate that we could separate survivors (green) from non-survivors, using only this small gene set, with a high degree of significance (permutation test, one-tailed P<0.0001; Figure 1B). In addition, we evaluated the cumulative ability of the gene set to distinguish survivors from non-survivors, based on individual gene traits, using leave-one-out cross-validation. This approach tests the robustness of a classifier, by using one sample as a testing set and the remaining samples as a training set, then classifying the left-out sample based on the training set. This procedure is repeated for all samples, and the robustness of a classifier is evaluated by a receiving operator characteristic (ROC) curve [57]–[59]. Leave-one-out cross-validation showed that our classifier correctly classified samples with 100% accuracy, i.e. no survivors were mistakenly classified as non-survivors, or vice versa (AUC = 1.00; Figure 1C).

### Identification of general survival-associated transcriptional profile

Although a minimal gene set can be useful for identifying individual genes associated with survival, we were also interested to know whether there was a general transcriptional profile associated with survival. In particular, we were interested to know if NHPs that survived EBOV infection displayed coordinated enrichment of specific signaling pathways or transcriptional responses. To identify a broad, survival-associated transcriptional profile that was also associated with anticoagulant treatment, we compared the expression profiles of anticoagulant-treated NHPs that survived EBOV infection (ETS) to NHPs that did not receive anticoagulant treatment (EO) and to NHPs that received anticoagulant treatment but did not survive EBOV infection (ETNS). We compared survivors to the two non-survivor groups (EO or ETNS) on Day 3, Day 6, and Days 3 and 6 together; probes were chosen according to the previously described criteria (see *Materials and Methods*).

**Figure 2:**
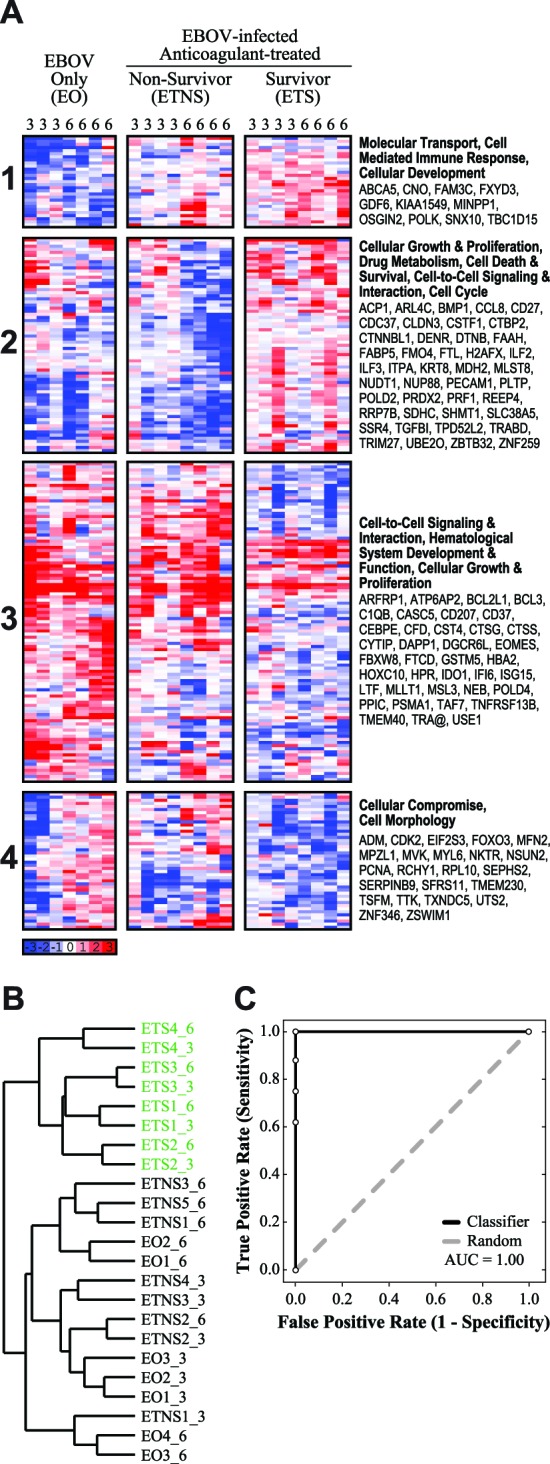
Survival following anticoagulant treatment is associated with a broad transcriptional profile. (**A**) A heatmap depicting the gene expression values of 245 probes (238 genes) which were found to distinguish between “EBOV-infected, Treated Survivors” (ETS), “EBOV-infect, Treated Non-Survivors”, and NHPs that did not receive anticoagulant treatment (“EBOV Only”; see *Materials and Methods*). The days post-infection are labeled at the top of each column. Blue indicates down-regulation, and red indicates up-regulation, of genes compared to pre-infection baseline; white indicates no change in expression (scale indicated). Probes are grouped into 4 clusters, denoted by numbers to the left of the heatmap. Functional annotation of each cluster is listed in bold to the right of the heatmap, along with a list of representative genes associated with the annotation. (**B**) A dendrogram depicting hierarchical clustering of individual samples, using the gene set of 245 probes (permutation test, one-tailed *P*<0.0001). ETS samples are indicated in green; samples are labeled “groupX_Y”, where X is the sample number and Y is the days post-infection. (**C**) A ROC curve depicting how accurately this gene set can classify survivors and non-survivors, based on leave-one-out cross-validation (AUC = 1.00). This gene set is indicated in black, and a random ROC curve is indicated in grey.

Using this approach, we identified a total of 245 unique probes, corresponding to 238 annotated genes, which accurately differentiated the three groups (Figure 2). Hierarchical clustering of the 245 probes showed that the probes clustered into four gene expression patterns: (1) down-regulation in the EO group on both days, compared with up-regulation in survivors on both days; (2) down-regulation in the ETNS group on Day 6, compared with up-regulation in survivors on both days; (3) up-regulation in the non-survivor groups on both days, compared with down-regulation in survivors on both days; and, (4) up-regulation in the non-survivor groups on Day 6, compared with down-regulation in survivors on both days (Figure 2A). In general, the overall pattern of these separate clusters is one of significant and opposing regulation of expression when comparing NHPs that survived EBOV infection to NHPs that did not survive infection, regardless of treatment.

Analysis of the overall gene set using Ingenuity Pathway Analysis (IPA; Ingenuity Systems) identified several statistically significant cellular and molecular functions, including cell death and survival, cellular growth and proliferation, infectious disease, cell cycle, and immune cell trafficking. In addition, we probed the 4 gene clusters to determine if there were any cluster-specific pathways or networks that were functionally enriched. We found that the four main clusters were associated with: (1) molecular transport, cell-mediated immune response, and cell development; (2) cellular growth and proliferation, drug metabolism, cell death and survival, cell-to-cell signaling and interaction, and cell cycle; (3) cell-to-cell signaling and interaction, hematological system development and function, and cellular growth and proliferation; and, (4) cellular compromise, and cell morphology (Figure 2A). In particular, we observed that many of the genes in Cluster 2 were associated with cell metabolism, suggesting that this process is down-regulated in non-survivors when compared to survivors. Cluster 3 is also heavily associated with immune and inflammatory responses, and several genes are expressed predominantly by immune cells. These functional annotations are consistent with previous studies assessing host immune response to EBOV infection using microarrays [22], [24]. For example, we observed up-regulation of genes associated with innate immune response, regulation of cytokine and chemokine production, regulation of apoptosis, and interferon response, as reported in previous studies [22], [24]. The observed regulation of these genes is consistent with the hypothesis that non-survivors experience severe dysregulation of the immune response, as opposed to survivors, which maintain a normal level of expression [22], [24].

To evaluate the classification of our gene set, we hierarchically clustered individual arrays ([37]; *complete linkage* method; Figure 2B). The resulting dendrogram has two major branches, in which NHPs that survive EBOV infection (green) cluster separately from those that did not (black; Figure 2B), indicating that the expression profiles of survivors is distinguishable from the expression profile of non-survivors. Correspondingly, this gene set allowed us to accurately distinguish between the survivors and the combined non-survivor groups (permutation test, one-tailed P<0.0001); however, we found no significant difference in the expression profiles when comparing the two non-survivor groups. Additionally, this gene set is capable of perfectly classifying survivor and non-survivor groups (Figure 2C). As in Figure 1, we used leave-one-out cross-validation to evaluate whether survivors could be distinguished from non-survivors. We found that cross-validation was able to correctly classify samples with 100% accuracy, i.e. no survivors were mistakenly classified as non-survivors, or vice versa (AUC = 1.00; Figure 2C). These two tests confirm that the expression profiles of survivors are distinguishable from the expression profile of non-survivors; however, the expression profile of untreated non-survivors (EO) is indistinguishable from that of treated non-survivors (ETNS). Finally, we compared this list of 238 genes against the previously identified list of 20 probes which were highly predictive for distinguishing between survivors and non-survivors. Of the 20 probes, we found that 17 (85%) also appeared in the set of 238 genes. These results confirm that the 238 genes are predictive for distinguishing between treatment groups and survival outcomes, and that the predictive power is comparable to the list of 20 highly predictive genes.

### Survival-associated genes are transcriptionally interconnected

To further investigate how these survival-associated genes are functionally associated, we determined if the genes were transcriptionally related, *e.g.* by being participants in a major pathway or signaling network, or by having a common upstream transcriptional regulator. We used IPA to probe the set of 238 genes in order to identify transcriptional regulators that were common to a large number of genes in our dataset.

**Figure 3:**
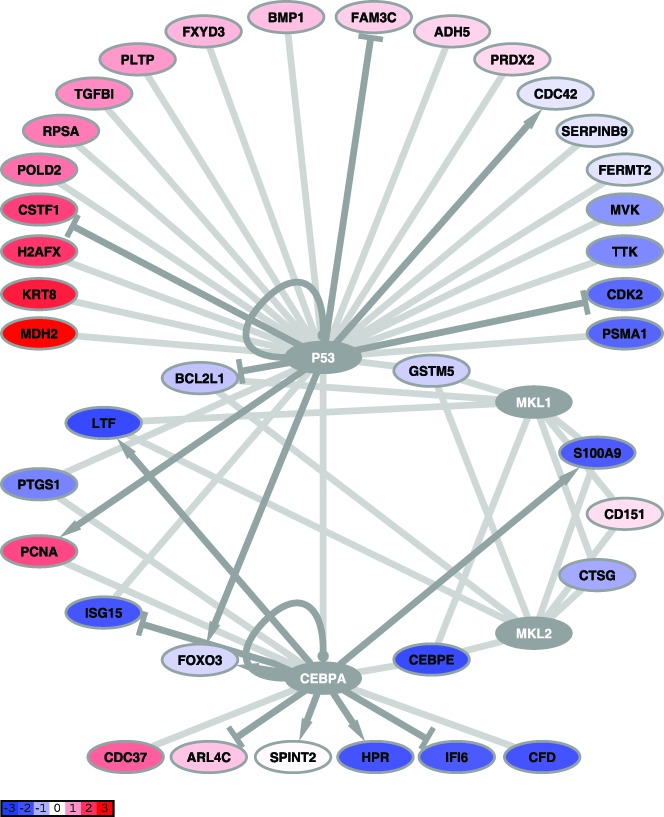
Survival-associated genes are transcriptionally interconnected. A network illustrating transcriptional connections between 37 genes which distinguish between “EBOV-infected, Treated Survivors” (ETS), “EBOV-infect, Treated Non-Survivors” (ETNS), and NHPs that did not receive anticoagulant treatment (“EBOV Only”; see *Materials and Methods*). Nodes are labeled with the gene name, edges indicate regulation of expression from transcription factor to target gene; arrows indicate up-regulation, bars indicate down-regulation, circles indicate up- and down-regulation, and unmarked, light grey edges indicate unknown regulation. Nodes are colored according to the difference in mean expression between the ETS and “Non-Survivor” (NS; ETNS and EO) groups; blue indicates lower expression values in the ETS group compared to the NS group, red indicates higher expression values in the ETS group compared to the NS group (scale indicated). Nodes that are colored grey (*e.g.* p53) are master transcriptional regulators which were not identified as being differentially expressed by our protocol.

This analysis identified four transcription factors whose downstream targets were statistically over-represented in our network: CCAAT/enhancer-binding protein alpha (CEBPA; Fisher's exact test, *P*<0.0001), tumor protein 53 (p53; *P*<0.001), megakaryoblastic leukemia 1 (MKL1; *P*<0.0001) and myocardin-like protein 2 (MKL2; *P*<0.0001). The probability of finding at least as many up-stream transcriptional regulators in a gene set of 245 probes is extremely unlikely, suggesting that this result is not due to random chance (re-sampling, *P*≈0.01). The transcription factors and their targets are shown in Figure 3, which shows genes colored according to the difference in mean expression between NHPs that survived EBOV infection and those that did not. This network of transcription factors and their downstream targets is highly interconnected, with several transcription factors sharing targets; in particular, MKL1 and MKL2 appear to co-regulate all their targets (Figure 3). As would be expected for transcription factors that are post-translationally activated, the four transcription factors were not themselves significantly differentially regulated in our dataset. For each transcription factor, we compared the expression of the downstream targets in NHPs which survived infection to NHPs that did not survive infection, to determine if downstream targets correlated with survival.

### CCAAT/enhancer binding protein alpha (CEBPA)-regulated genes are down-regulated in NHPs that survive EBOV infection when compared to non-survivors

**Figure 4:**
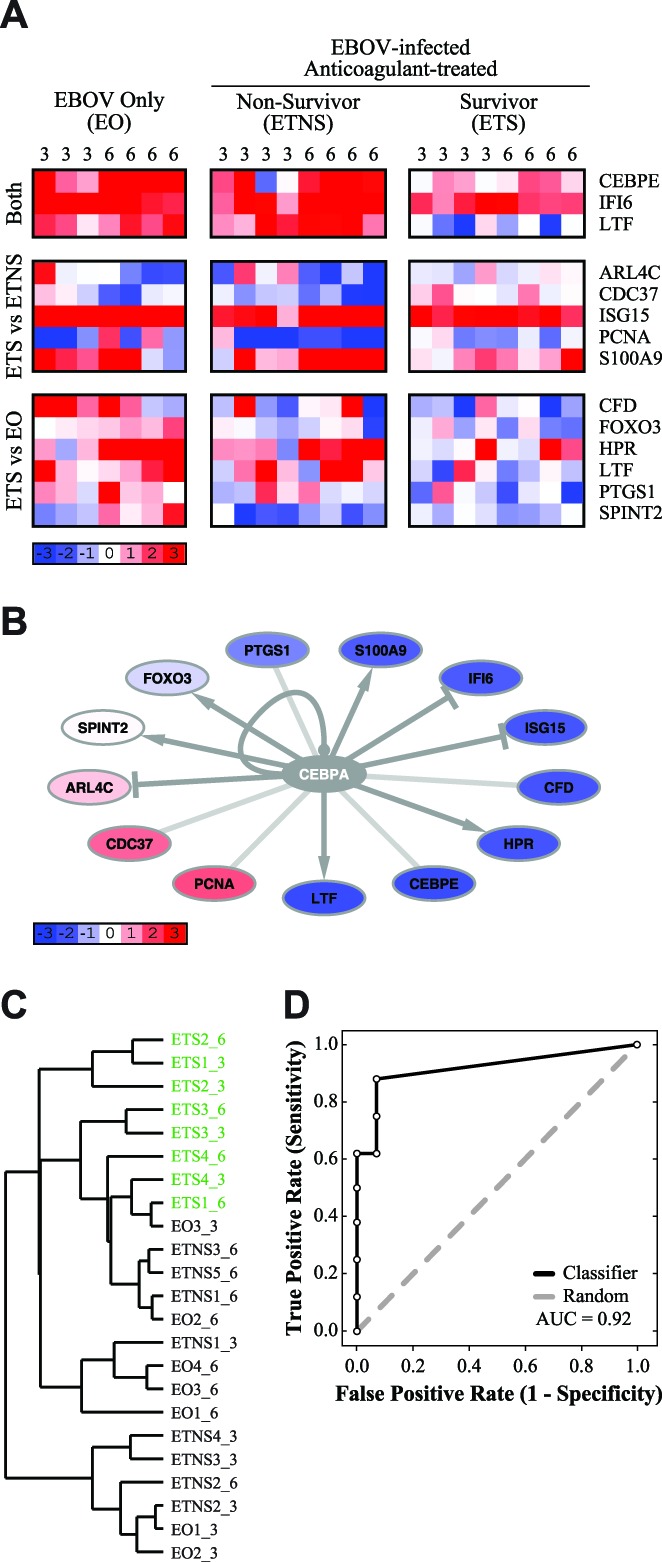
CCAAT/enhancer binding protein alpha (CEBPA)-regulated genes are down-regulated in NHPs that survive EBOV infection when compared to non-survivors. (**A**) A heatmap depicting the gene expression values of 14 probes (13 genes) which are transcriptionally regulated by CEBPA. The days post-infection are labeled at the top of each column. Probes are grouped according to which comparison identified them, listed to the left. Gene names are listed to the right. Blue indicates down-regulation, and red indicates up-regulation, of genes compared to pre-infection baseline, white indicates no change in expression (scale indicated). (**B**) A network illustrating transcriptional connections between the 14 genes and CEBPA. Blue indicates lower expression values in “EBOV-infected, Treated Survivors” (ETS), compared to “Non-Survivors” (NS), red indicates higher expression values in the ETS group compared to the NS group (scale indicated). Grey nodes (*e.g.* CEBPA) were not identified as being differentially expressed by our protocol. (**C**) A dendrogram depicting hierarchical clustering of individual samples, using the gene set of 14 probes. ETS samples are indicated in green; samples are labeled “groupX_Y”, where X is the sample number and Y is the days post-infection. (**D**) A ROC curve depicting how accurately this gene set can classify survivors and non-survivors, based on leave-one-out cross-validation (AUC = 0.92). This gene set is indicated in black, and a random ROC curve is indicated in grey.

Of the 238 genes associated with a transcriptional pattern correlated with survival, 13 genes are transcriptionally regulated by CEBPA (Figure 4). Of these, 2 probes (CEBPE, and IFI6) distinguished NHPs that survived infection (“EBOV-infected, Treated Survivors”; ETS) from those that did not (“Non-Survivors”; NS). Both probes had lower expression values in the ETS group. Five probes distinguished between survivors and “EBOV-infected, Treated Non-Survivors” (ETNS); 3 of these probes had higher expression values in survivors (ARL4C, CDC37, and PCNA), and 2 had lower expression values in survivors (ISG15 and S100A9). Six probes distinguished between survivors and the “EBOV Only” (EO) group, of which 4 had lower expression values in survivors (CFD, FOXO3, HPR, and PTGS1). One probe, SPINT2, distinguished between survivors and the EO group only on Day 6. One gene, LTF, was identified by two non-identical probes; one probe distinguished survivors from both non-survivor groups, whereas the other probe only distinguished between survivors and the “EBOV Only” group. In both cases, the probe had lower expression values in survivors (Figure 4A).

To confirm this expression pattern, we used RT-PCR to examine a subset of the CEBPA-regulated genes (data not shown). We examined 11 of the 13 genes by comparing the changes in expression on Days 3 and 6 in the microarray dataset to the RT-PCR dataset (see *Materials and Methods*). The RT-PCR dataset reflected the trends observed in the microarray dataset with above 60% accuracy in all three treatment groups (EO: 68.2%; ETNS: 77.3%; ETS: 88.6%). For CEBPA-regulated genes, the RT-PCR confirms the expression trends observed in the microarray dataset.

The observed expression patterns of the downstream targets are consistent with decreased transcriptional activity of CEBPA (Figure 4B), when compared to a previous study which identified transcriptional targets of CEBPA (GEO accession GSE2188, [60]). Although there is a clear pattern of expression that suggests an underlying biological mechanism (*i.e.* down-regulation of CEBPA), this set of probes alone is insufficient to statistically distinguish between NHPs that survived EBOV infection and those that did not. Hierarchical clustering of CEBPA target expression patterns in individual arrays reveals a dendrogram in which the survivor and non-survivor groups are not discrete (Figure 4C; ETS indicated in green). There is some similarity between the expression profiles of survivors (green) and non-survivors, especially treated non-survivors (ETNS), which appears to be driven by within-animal responses, and not by treatment or survival (Figure 4C). However, we found that leave-one-out cross-validation was able to correctly classify samples with high accuracy, i.e. very few survivors were mistakenly classified as non-survivors (AUC = 0.92; Figure 4D). This result demonstrates that, when considering the CEBPA signature as a classifier, survivors are distinguishable from non-survivors, but with less accuracy than the full gene sets identified in Figures 1 and 2.

### Tumor protein 53 (p53) regulates transcription of some survival-associated genes

**Figure 5:**
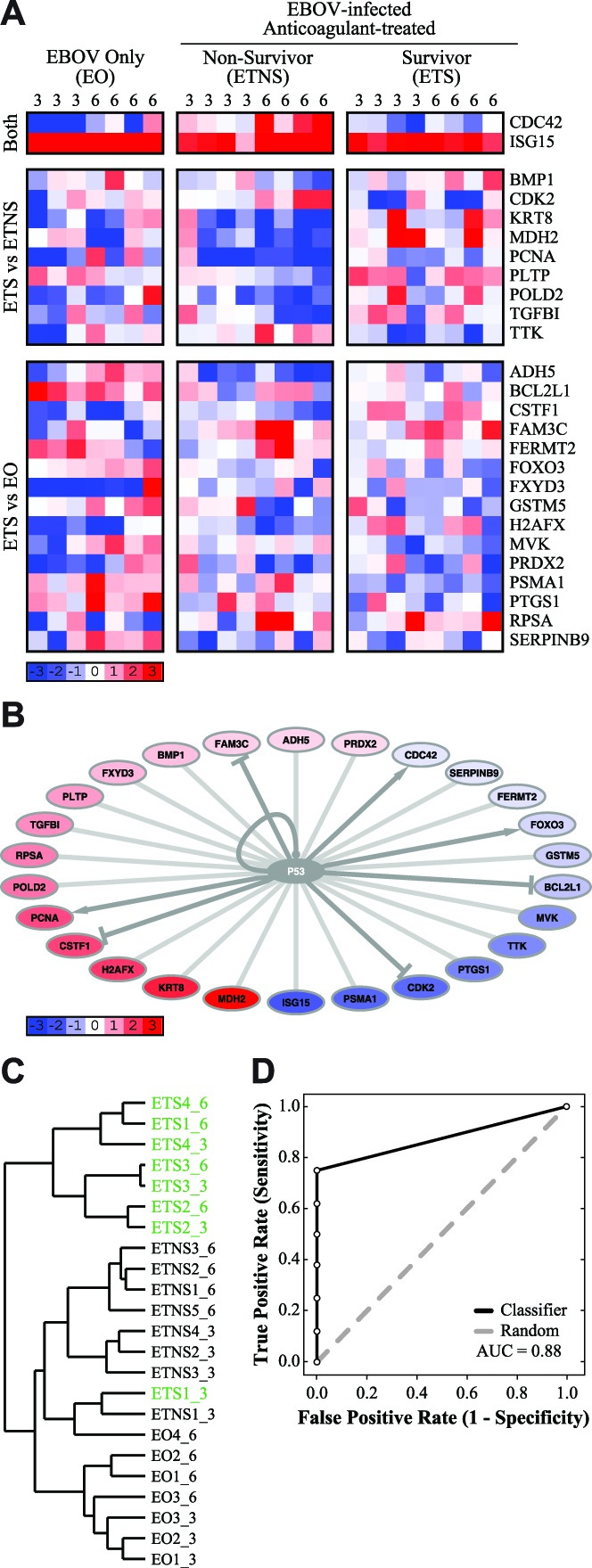
Tumor protein 53 (p53) regulates transcription of some survival-associated genes. (**A**) A heatmap depicting the gene expression values of 26 probes (26 genes) which are transcriptionally regulated by p53. The days post-infection are labeled at the top of each column. Probes are grouped according to which comparison identified them, listed to the left. Gene names are listed to the right. Blue indicates down-regulation, and red indicates up-regulation, of genes compared to pre-infection baseline, white indicates no change in expression (scale indicated). (**B**) A network illustrating transcriptional connections between the 26 genes and p53. Blue indicates lower expression values in “EBOV-infected, Treated Survivors” (ETS), compared to “Non-Survivors” (NS), red indicates higher expression values in the ETS group compared to the NS group (scale indicated). Grey nodes (*e.g.* p53) were not identified as being differentially expressed by our protocol. (**C**) A dendrogram depicting hierarchical clustering of individual samples, using the gene set of 26 probes (permutation test, one-tailed P<0.0001). ETS samples are indicated in green; samples are labeled “groupX_Y”, where X is the sample number and Y is the days post-infection. (**D**) A ROC curve depicting how accurately this gene set can classify survivors and non-survivors, based on leave-one-out cross-validation (AUC = 0.88). This gene set is indicated in black, and a random ROC curve is indicated in grey.

We identified 26 probes, corresponding to 26 genes, which are transcriptionally regulated by p53 (Figure 5A). Of these, 2 probes (CDC42 and ISG15) distinguished NHPs that survived EBOV infection (“EBOV-infected, Treated Survivors”; ETS) from those that did not (“Non-Survivors”; NS); in addition, both probes had lower values in survivors. Nine probes distinguished between survivors and “EBOV-infected, Treated Non-Survivors” (ETNS), of which 7 had higher expression in survivors (BMP1, KRT8, MDH2, PCNA, PLTP, POLD2, and TGFBI), and 2 had lower expression values in survivors (CDK2 and TTK). Fifteen probes distinguished between survivors and the “EBOV Only” (EO) group, of which 7 had higher expression values in survivors (ADH5, CSTF1, FAM3C, FXYD3, H2AFX, PRDX2, and RPSA), and 8 had lower expression values in survivors (BCL2L1, FERMT2, FOXO3, GSTM5, MVK, PSMA1, PTGS1, and SERPINB9; Figure 5B). However, the observed expression patterns of these targets were not consistent with any clearly defined activation or repression of p53, suggesting that the transcription factor may be differently regulated in different cell types within the PBMC population.

To confirm this expression pattern, we used RT-PCR to examine a subset of the P53-regulated genes (data not shown). We examined 20 of the 26 genes (76.9%) by comparing the changes in expression on Days 3 and 6 in the microarray dataset to the RT-PCR dataset (see *Materials and Methods*). The three treatment groups had comparable levels of agreement between the RT-PCR dataset and the microarray dataset (EO: 70%; ETNS: 72.5%; ETS: 80%). For P53-regulated genes, the RT-PCR confirms the expression trends observed in the microarray dataset.

In a comparison to an independently derived dataset examining the effects of p53 dosage, we found that approximately 50% of our p53 targets had an expression pattern concordant with p53 activation, whereas the other 50% had discordant expression patterns which suggested inhibition or down-regulation of p53 (GEO accession GSE11547, Hosako *et al.*). Because a consensus pattern of expression could not be established, we cannot draw conclusions about the activity of p53 in NHPs that survived EBOV infection when compared to non-survivors. Despite this lack of consensus regarding the activity of p53 in different treatment groups, hierarchical clustering revealed that this set of probes is able to distinguish survivors and non-survivors with some accuracy (Figure 5C; ETS indicated in green; permutation test, one-tailed P<0.0001). The observed dendrogram has three major branches: (i) an ETS cluster, which is clearly separated from the other branches of the tree; (ii) an ETNS cluster with two misclassified arrays, which are EO and ETS samples; and, (iii) an EO cluster. This suggests that the gene set is sufficient to distinguish survivors from non-survivors, and secondarily can also distinguish the two non-survivor groups from one another. The case of a survivor array being misclassified as a non-survivor array is understandable, given that the array sample is from Day 3, when gene expression differences are relatively small between all different treatment groups. We found that leave-one-out cross-validation was able to correctly classify samples with some accuracy (AUC = 0.88; Figure 5D), although the classification was not as strong as the full dataset.

### Megakaryoblastic leukemia 1 (MKL1) and myocardin-like protein 2 (MKL2) jointly regulate some survival-associated genes

**Figure 6:**
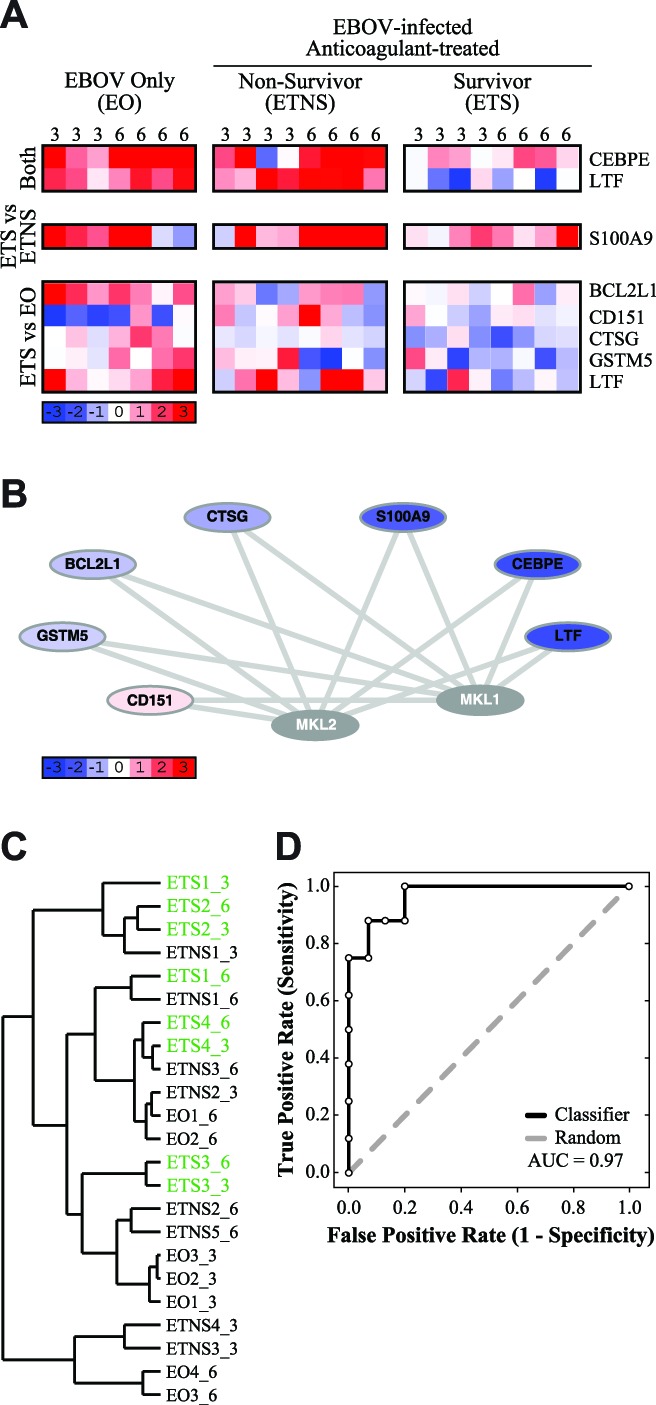
Megakaryoblastic leukemia 1 (MKL1) and myocardin-like protein 2 (MKL2) jointly regulate some survival-associated genes. (**A**) A heatmap depicting the gene expression values of 8 probes (7 genes) which are jointly transcriptionally regulated by MKL1 and MKL2. The days post-infection are labeled at the top of each column. Probes are grouped according to which comparison identified them, listed to the left. Gene names are listed to the right. Blue indicates down-regulation, and red indicates up-regulation, of genes compared to pre-infection baseline, white indicates no change in expression (scale indicated). (**B**) A network illustrating transcriptional connections between the 7 genes and MKL1/2. Blue indicates lower expression values in “EBOV-infected, Treated Survivors” (ETS), compared to “Non-Survivors” (NS), red indicates higher expression values in the ETS group compared to the NS group (scale indicated). Grey nodes (*e.g.* MKL1) were not identified as being differentially expressed by our protocol. (**C**) A dendrogram depicting hierarchical clustering of individual samples, using the gene set of 7 probes. ETS samples are indicated in green; samples are labeled “groupX_Y”, where X is the sample number and Y is the days post-infection. (**D**) A ROC curve depicting how accurately this gene set can classify survivors and non-survivors, based on leave-one-out cross-validation (AUC = 0.97). This gene set is indicated in black, and a random ROC curve is indicated in grey.

Finally, we identified 8 probes, corresponding to 7 genes, whose transcription is jointly regulated by MKL1 and MKL2 (Figure 6). Of these, 2 probes (CEBPE and LTF) distinguished NHPs that survived EBOV infection (“EBOV-infected, Treated Survivors”; ETS) from those that did not (“Non-Survivors”; NS). Notably, CEBPE is also co-regulated by another member in the CCAAT-enhancer binding protein family, CEBPA; therefore, it is included in both gene sets. In addition, 1 probe (S100A9) distinguished between survivors and “EBOV-infected, Treated, Non-Survivors” (ETNS), and 5 probes distinguished between survivors and the “EBOV Only” group (EO; BCL2L1, CD151, CTSG, GSTM5, and LTF). All genes had lower expression values in the survivors than non-survivors (Figure 6A), with the exception of CD151, which exhibited significant down-regulation in the EO group. Importantly, MKL1 and MKL2 appear to co-regulate the full set of genes (Figure 6B). Notably, the majority of the genes regulated by MKL1 and MKL2 are also regulated by CEBPA (CEBPE, LTF and S100A9) or p53 (BCL2L1 and GSTM5), suggesting that the regulatory pattern exhibited by these genes could be confounded by regulatory activity from additional transcription factors.

We confirmed the expression pattern of the 7 genes regulated by MKL1 and MKL2 by RT-PCR (data not shown). We examined all genes co-regulated by these transcription factors by comparing the changes in expression on Days 3 and 6 in the microarray dataset to the RT-PCR dataset (see *Materials and Methods*). For both non-survivor groups, 71.4% of genes exhibited the same expression trends in both the microarray dataset and the RT-PCR dataset. For survivors, 85.7% of genes exhibited the same expression trends in both the microarray dataset and the RT-PCR dataset. For MKL1 and MKL2-regulated genes, the RT-PCR confirms the expression trends observed in the microarray dataset.

A previous study found that a double-knockout of MKL1 and MKL2 increases expression of CEBPE, CTSG, GSTM5, LTF and S100A9, suggesting that MKL1 and MKL2 may jointly act as repressors for these genes under natural conditions [61]. In contrast, a double-knockout of MKL1 and MKL2 decreases expression of BCL2L1 and CD151, suggesting that MKL1 and MKL2 activate transcription of these genes [61]. The expression pattern observed with this gene set suggests that MKL1 and MKL2 may be up-regulated or activated in NHPs that survive EBOV infection, compared to those that do not. Although this observed expression pattern is consistent with what would be observed if MKL1 and MKL2 were activated, this set of probes is too small to distinguish between survivors and non-survivors groups (Figure 6C; ETS indicated in green). A hierarchically-clustered dendrogram has two major branches, which are each interspersed with EO, ETNS and ETS samples; this suggests that the overall expression profile of survivors and non-survivors is not statistically distinguishable. Although the dendrogram suggests that the overall expression profile in each array is incapable of distinguishing survivors and non-survivors, evaluation of individual gene contributions using leave-one-out cross-validation shows that this gene set is capable of classifying samples with high accuracy (AUC = 0.97; Figure 6D).

## Discussion

These results show the potential of high-throughput transcriptional studies for identifying putative markers of survival following EBOV infection. In particular, we identified a minimal survival-associated gene set that accurately distinguished survival outcome following post-infection anticoagulant treatment of non-human primates (NHPs) infected with EBOV. We identified 20 genes that were characterized by significant, coherent and opposing expression patterns when comparing survivors and non-survivors. Several of these genes exhibit differential regulation as early as 3 days post-infection, prior to the appearance of clinical symptoms of EBOV infection; this early differential regulation is especially important for the identification of early-stage biomarkers to distinguish disease outcomes.

Importantly, several of these genes are associated with different viral infections [46]–[50]. Proteins such as ILF3 and RUVBL2 are known to suppress viral replication in other viruses [44], [45], [53], and we observe that their expression is higher in survivors than non-survivors. Notably, an isoform of ILF3 is known to bind EBOV protein VP35, suppressing the function of the viral polymerase [54]. This suggests a mechanism of action in which survivors may up-regulate the transcription of certain genes, *e.g.* ILF3, in order to suppress viral replication.

We also observed that microRNA 122 (miR-122) is down-regulated in survivors compared to non-survivors, suggesting that inhibition of miR-122 activity increases survival following EBOV infection. To date, there have been no studies investigating whether miR-122 interacts with the EBOV genome, but it is well-documented that miR-122 binds the Hepatitis C virus genome to support the replication of this virus [55], [56]. Comparison of the putative binding motifs of miR-122 [55] to the consensus sequence of EBOV (Mayinga, Zaire, 1976) [62] reveals multiple potential binding sites in the viral genome (data not shown). This suggests that miR-122 is worth further investigation as a regulator of EBOV infection.

Though a minimal set of 20 genes could separate survivors from non-survivors, we were interested in also studying the general host response to EBOV infection, and to determine if there was a survival-associated transcriptional profile. We identified 238 genes that accurately distinguished treatment groups and survival outcomes following EBOV infection. Functional annotation of these 238 genes confirmed that this gene set was comparable with previously published studies of EBOV infection [21], [22], [24], [27]. In particular, the expression pattern that we observe for IL6, in which non-survivors are significantly more up-regulated in early stages of infection than survivors, is supported by similar changes in protein concentration reported in previous studies [7], [9], [25]. There is also a pattern of significant up-regulation of genes associated with immune response in non-survivors, but not in survivors, consistent with the hypothesis that non-survivors exhibit severe dysregulation of the inflammatory response [21], [22], [24], [27]. Importantly, our work highlights the utility of using a minimal survival-associated gene set to identify individual genes correlated with survival following EBOV infection, which is not possible when assessing global transcriptional responses in the host, as in previous work [22], [24].

When we compare our data to the results of a study of survival-associated biomarkers in human samples from the Sudan virus (SUDV) outbreak [25], we find notable similarities. Similar to this study, we observed significant up-regulation chemokines and cytokines, such as CCL3, CXCL10, IL1RN, IL6 and TNF, throughout infection. This study reported that ferritin was a good correlate of hemorrhage and death in response to SUDV infection [25]. We observed down-regulation of ferritin throughout infection, although this pattern was not correlated with survival on a transcriptional level. However, we find that another iron-binding protein, lactotransferrin (LTF) is highly correlated with survival outcome in EBOV-infected NHPs. This similarity suggests that iron modulation may play an important role in regulating filovirus infection, especially in relation to coagulopathies and hemorrhage, and that our biomarkers merit further study in a human system.

We identified 3 transcriptional modules which were significantly enriched in the gene set: (i) CCAAT/enhancer-binding protein alpha (CEBPA); (ii) tumor protein 53 (p53); and (iii) megakaryoblastic leukemia 1 (MKL1) and myocardin-like protein 2 (MKL2). Previous studies have shown that p53 plays a crucial role during viral infection, which invariably disrupts normal cell cycle processes, in a variety of DNA and RNA viruses [63]–[66]. In particular, p53 is known to be associated with the Type I interferon response and has been previously reported to enhance viral-induced apoptosis in other infections [65], [67]. In our examination of p53-regulated genes, we found that several are associated with regulation of apoptosis (e.g. BCL2L1 [68], CDC42 [69], CDK2 [70], FOXO3 [71], and PCNA [72]). This may suggest a role for p53 as a mediator of apoptosis following EBOV infection. However, due to a lack of a consensus pattern of expression, we are unable to determine the underlying regulation of p53 in this dataset, and therefore cannot draw conclusions about the activity of p53 in NHPs that survived EBOV infection when compared to non-survivors.

Interestingly, there is no consensus as to how CCAAT/enhancer binding proteins (such as CEBPA) function in general during viral infection, but they have been previously implicated in promoting the replication of some viruses. For example, CEBP binding sites exist in the Human immunodeficiency virus (HIV) genome, and CEBPs are required for the replication and regulation of HIV [73]–[75] and Simian immunodeficiency virus [76]. Similarly, physical binding and interactions have been observed between CEBPs and the proteins of Hepatitis B virus [77], [78], Epstein-Barr virus [79], and HIV [80]. In contrast, CEBPs have been known to down-regulate or inhibit replication of T-cell leukemia virus [81], [82] and some human papillomaviruses [83]. Our results suggest that strong CEBP responses are correlated with poorer prognosis following EBOV infection. We hypothesize that CEBP-regulated genes may contribute to the inflammatory response to infection, or to the dysregulation of coagulation.

Our studies are the first to suggest a role for MKL1 and MKL2 in viral infection, although roles for both proteins were recently identified in megakaryocyte differentiation and platelet formation [61]. Because dysregulation of coagulation is a common characteristic of EBOV infection, it is possible that MKL1 and MKL2 regulate coagulation in response to EBOV challenge. Indeed, we observe that the downstream targets of MKL1 and MKL2 exhibit an expression profile consistent with up-regulation of MKL1 and MKL2 in survivors, compared to non-survivors. This implies that survivors increase the regulation of coagulation processes, potentially avoiding the typical coagulopathies associated with late-stage EBOV infection. However, the majority of genes that are regulated by MKL1 and MKL2 are also regulated by CEBPA or p53, suggesting that the regulation observed is not due to the transcriptional activity of MKL1 and MKL2 alone. Despite this, these genes display a strong expression profile that is consistent with up-regulation of MKL1 and MKL2 when compared to a previous study [61], suggesting that survivors are able to recover in part due to normal MKL1 and MKL2 function.

It is important to note that we did not find a single unique gene that distinguished between survival outcomes of EBOV-infected NHPs, suggesting that survival following anticoagulant treatment is driven by a complex set of transcriptional responses. In addition, gene sets and pathways we have identified are associated with survival following anticoagulant treatment, and are therefore specific to this condition. We also stress that the observed results are in EBOV-infected NHPs, and our findings and conclusions may not be applicable to additional viral infections, although infection-specific signatures may exist. Under these conditions, we identify several complex transcriptional responses that clearly differentiate between survivors and non-survivors following EBOV infection. In particular, we observe several survival-associated profiles that are driven by specific upstream transcriptional regulators (*e.g.* CEBPA, p53, and MKL1/MKL2). Notably, these transcription factors have not been previously associated with EBOV infection, and would not have been identified without pathway analysis, due to lack of differential regulation. In particular, the ability of a small set of 20 genes to distinguish between survival outcomes suggests that they could potentially serve as biomarkers of disease outcome. Our results demonstrate that classification of treatment groups or disease outcome can be accomplished with a small gene set, which can be useful for identifying individual transcriptional markers associated with survival following anticoagulant treatment of EBOV infection.

## Supporting Information

Figure S1 This figure illustrates the overall process of microarray analysis described in this paper, including sample collection, microarray processing and normalization, and analysis of the data (as described in *Materials and Methods*).
(PNG)

Table S1 This table lists the 20 genes from the minimal survival-associated gene set (as described in *Materials and Methods*). The table lists the Gene Symbol, Gene Entrez ID, a brief description of the gene name, and a short list of gene functions as described by the Gene Ontology “function” category.
(XLS)

Table S2 This table lists 241 genes from the general survival-associated transcriptional profile (as described in *Materials and Methods*). The table lists the Gene Symbol, Gene Entrez ID, a brief description of the gene name, and a short list of gene functions as described by the Gene Ontology “function” category.
(XLS)
