# Supplementary figures and images for: Transcriptional Correlates of Disease Outcome in Anticoagulant-Treated Non-Human Primates Infected with Ebolavirus
Source: PLoS Negl Trop Dis. 2014 Jul 31;8(7):e3061. doi: 10.1371/journal.pntd.0003061 (PMC4117489; doi:10.1371/journal.pntd.0003061)

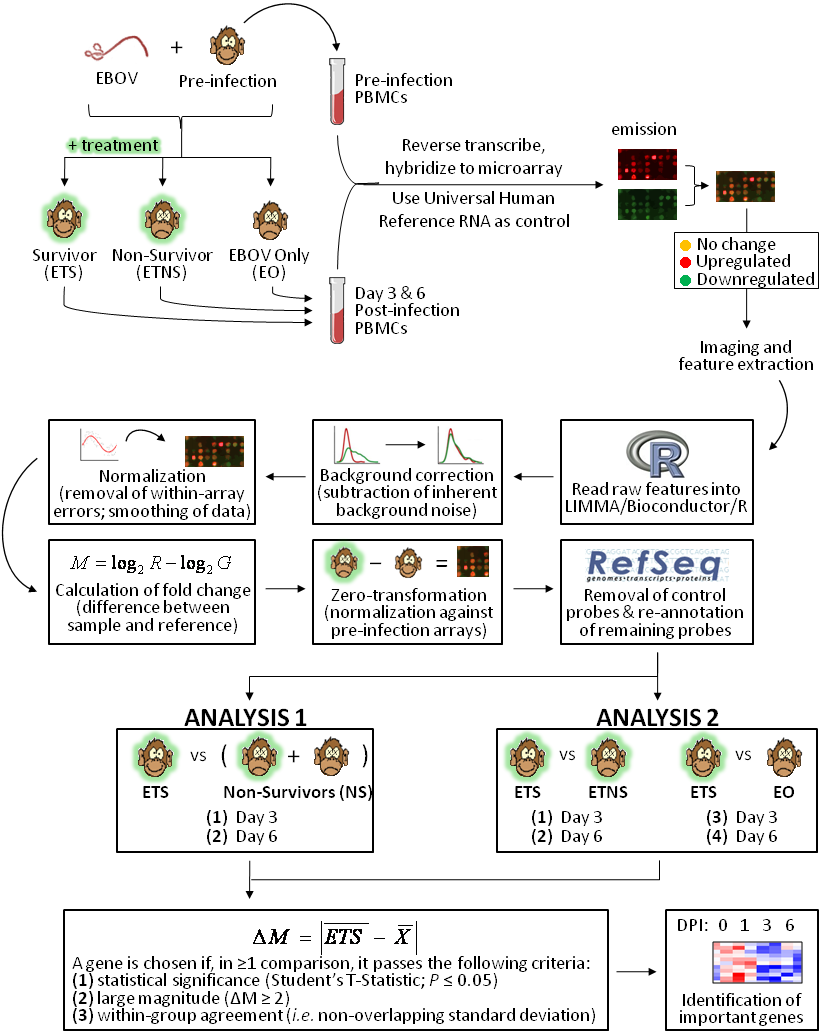

Supplement: Figure S1 — This figure illustrates the overall process of microarray analysis described in this paper, including sample collection, microarray processing and normalization, and analysis of the data (as described in Materials and Methods ). (PNG) [file pntd.0003061.s001.png]
